# Supplementary material for: Development of Multilayer Nanoparticles for the Delivery of Peptide-Based Subunit Vaccine against Group A Streptococcus
Source: Pharmaceutics. 2022 Oct 10;14(10):2151. doi: 10.3390/pharmaceutics14102151 (PMC9610843; doi:10.3390/pharmaceutics14102151)
Supplement: Supplementary file 1 [file pharmaceutics-14-02151-s001.zip › pharmaceutics-1946479-supplementary.pdf]

Development of Multi-Layer Nanoparticle for the Delivery of Peptide-Based Subunit Vaccine against Group A *Streptococcus*

Jolynn Kiong[1], Ummey Jannatun Nahar[1], Shengbin Jin[1], Ahmed O. Shalash[1], Jiahui Zhang[1], Prashamsa Koirala[1], Zeinab G. Khalil[2], Robert J Capon[2], Mariusz Skwarczynski[1], Istvan Toth[1,2,3], Waleed M. Hussein[1*]

[1] School of Chemistry and Molecular Biosciences, The University of Queensland, St Lucia, Queensland, Australia

[2] Institute for Molecular Bioscience, The University of Queensland, St Lucia, Queensland, Australia

[3] School of Pharmacy, The University of Queensland, Woolloongabba, Queensland, Australia

*Corresponding Author: Dr. Waleed M. Hussein, Email: w.hussein@uq.edu.au

Supplementary Data

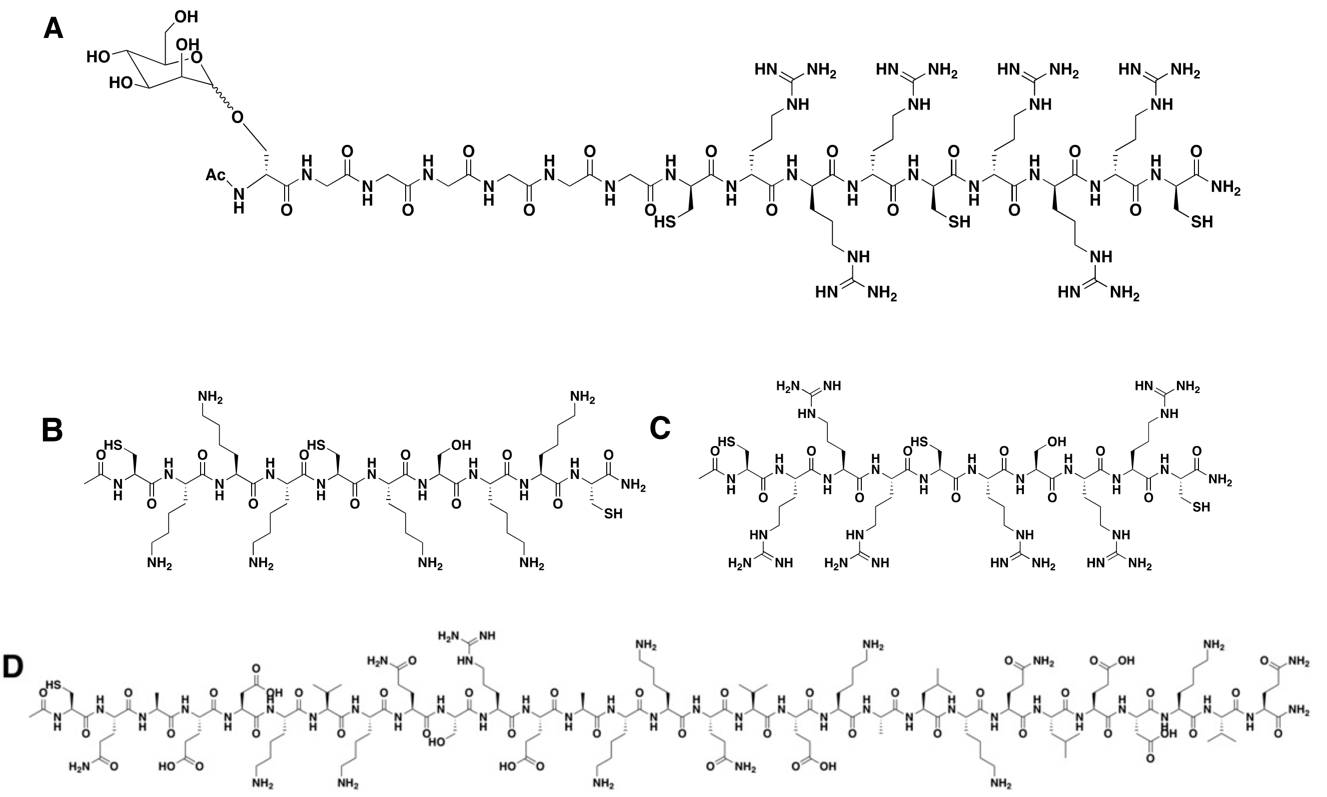

Figure S1. Chemical structures of four synthesised cationic peptides. **(A)** Mannosylated poly-Arg; molecular weight (MWT): 1895.87g/mol, purity ≥ 99% was achieved, yield 60.0% **(B)** Poly-Lys; MWT: 1224.8g/mol, purity ≥ 99% was achieved, yield 30.5% **(C)** Poly-Arg; MWT: 1392.7g/mol, purity ≥ 98% was achieved, yield 38.3% **(D)** J8-Cys; MWT: 3426.95g/mol, purity ≥ 98% was achieved, yield 55.5%

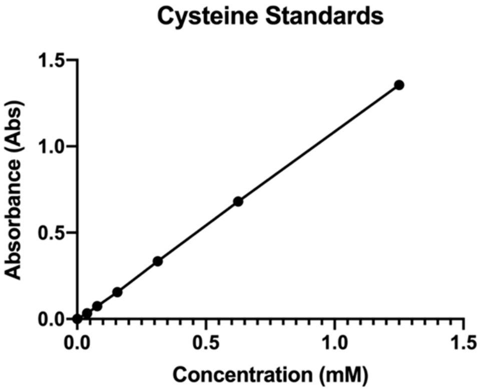

Oxidised Samples	Weight of sample (mg)	Concentration (mM)	Absorbance
Poly-Arginine	0.5mg in 0.75mL 10X PBS	0.417/ 0.208	0.001/0.00
Poly-Lysine	0.5mg in 0.83114mL 10X PBS		0.00/0.00

Figure S2. Ellman's Test for Quantification of Free Thiol. Cysteine standards of different concentration (varying from 0.0 to 1.25) were prepared to develop the cysteine standard curve to quantify the concentration of free thiols (left), polyArg and polyLys was prepared to a concentration of 0.417 and 0.208, and analysed to determine the number of free thiols after oxidation with ammonium bicarbonate solution (right)

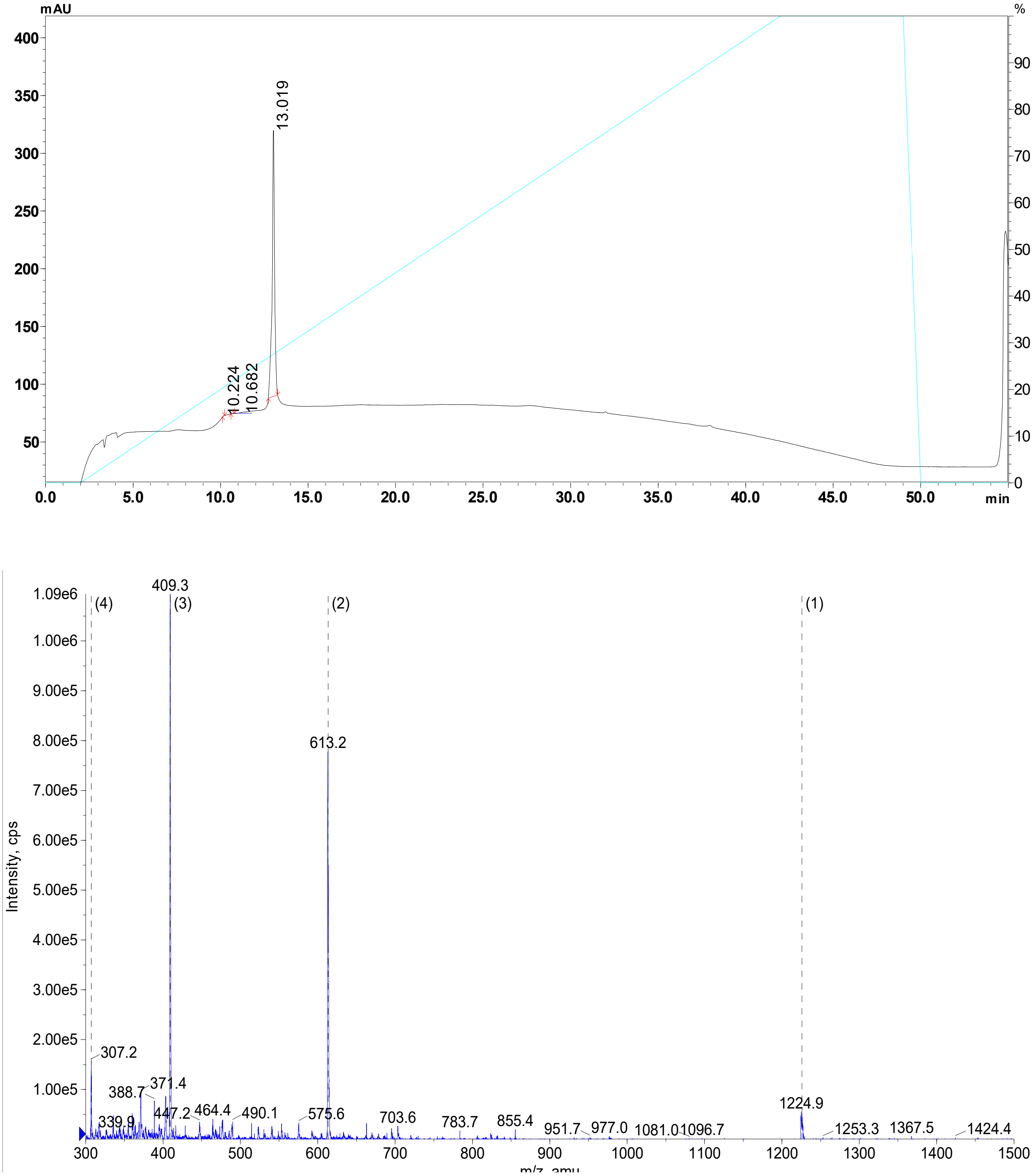

Figure S3. Analytical HPLC profile of poly-Lys (Ac-CKKKCKSKKC) (top); HPLC (C18): t_R = 13.0 min, purity ≥ 99%. Mol. Wt.= 1224.8g/mol. Mass spectrum of Poly-Lys (bottom): $[M+1H]^{1+}$: 1224.9 (calculated 1225.8), $[M+2H]^{2+}$: 613.2 (calculated 613.4), $[M+3H]^{3+}$: 409.3 (calculated 409.27); yield: 31%.

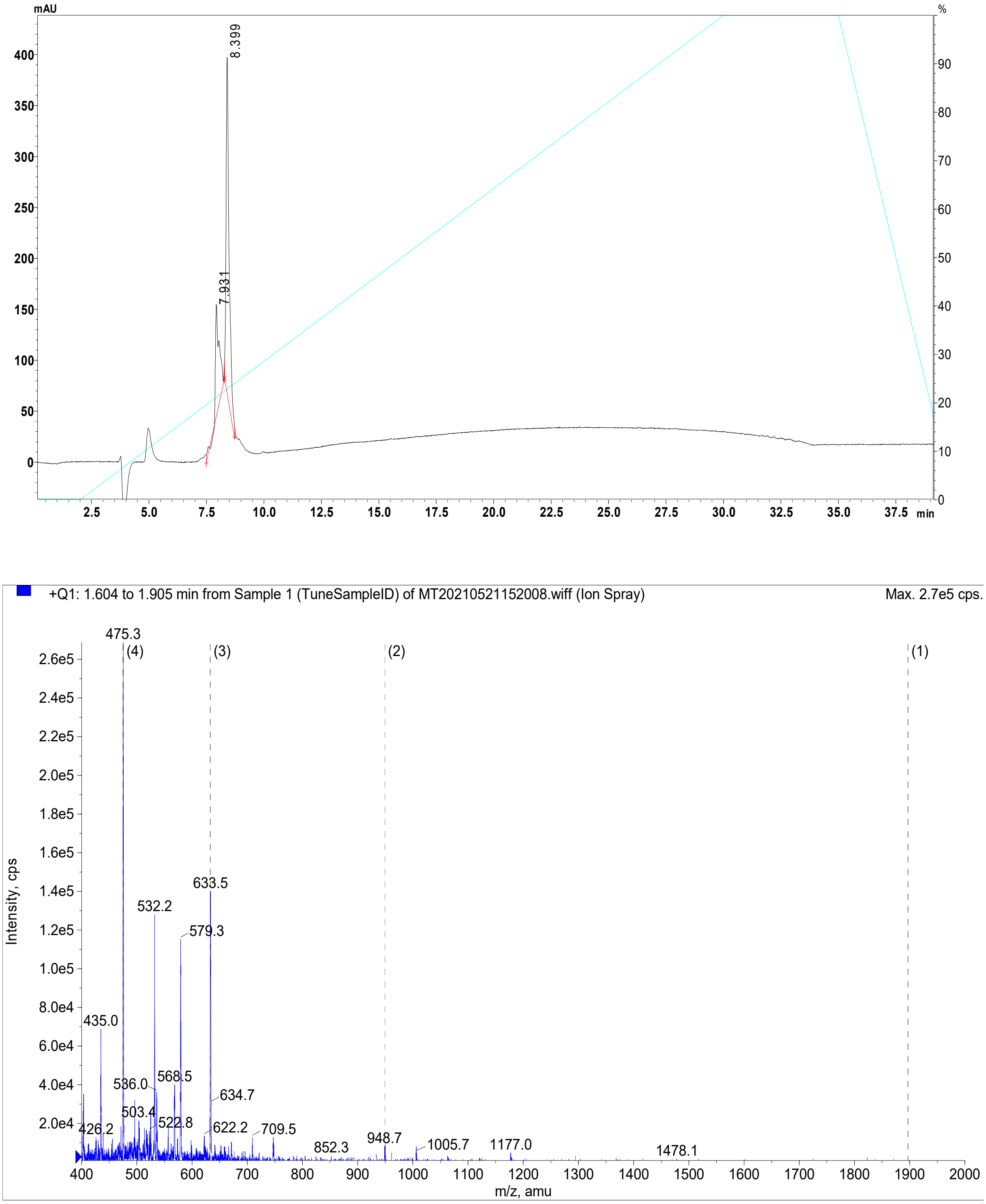

Figure S4. Analytical HPLC profile of α- and β- mannosylated poly-Arg (top); HPLC (C18): t_R = 7.9 and 8.4 min, purity ≥ 99%. Mol. Wt.= 1897.87g/mol. Mass spectrum of mannosylated poly-Arg (bottom): $[M+2H]^{2+}$: 948.7 (calculated 949.6), $[M+3H]^{3+}$: 633.5 (calculated 633.4), $[M+4H]^{4+}$: 475.3 (calculated 475.28); yield: 60%

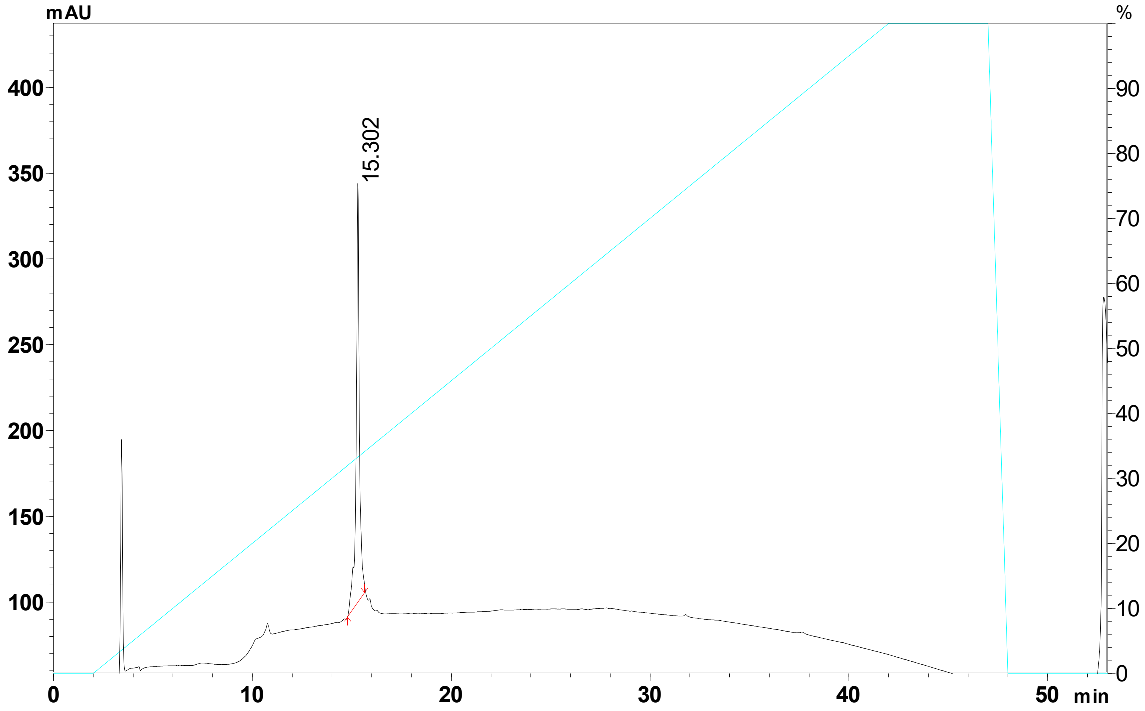

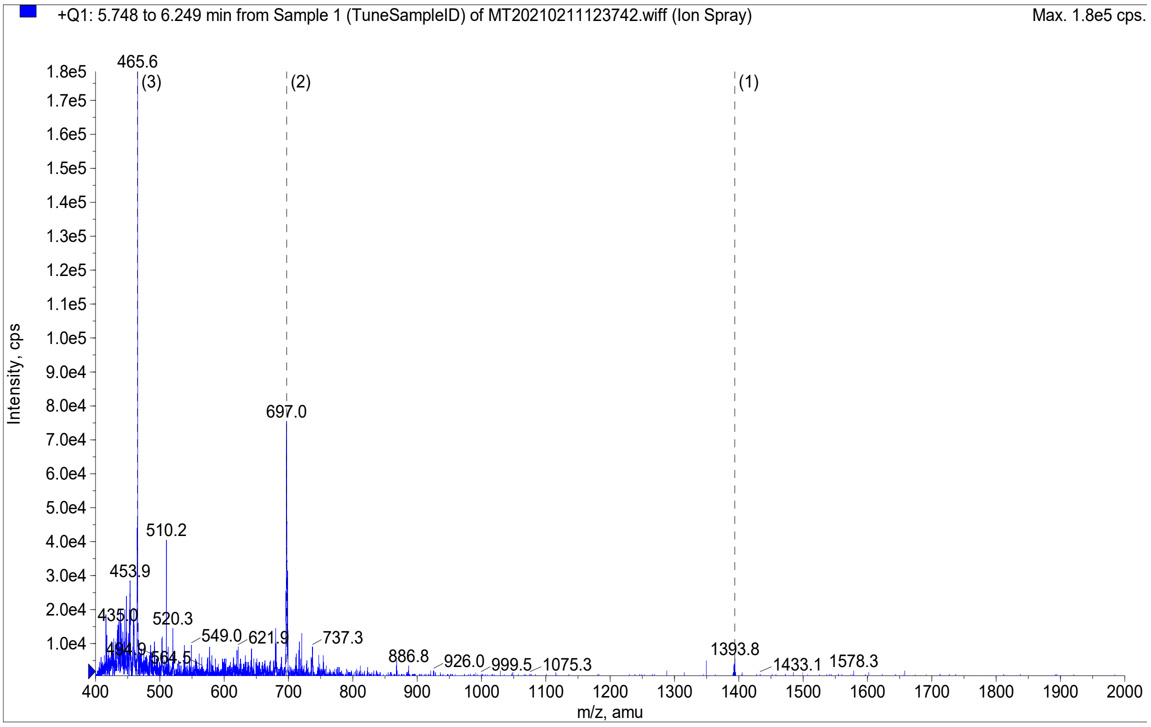

Figure S5. Analytical HPLC profile of poly-Arg (top); HPLC (C18): t_R = 15.3 min, purity ≥ 98%. Mol. Wt.= 1392.7g/mol. Mass spectrum of poly-Arginine (bottom): $[M+1H]^{1+}$: 1393.8 (calculated 1393.7) , $[M+2H]^{2+}$: 697.0 (calculated 697.4), $[M+3H]^{3+}$: 465.6 (calculated 465.2); yield: 38%

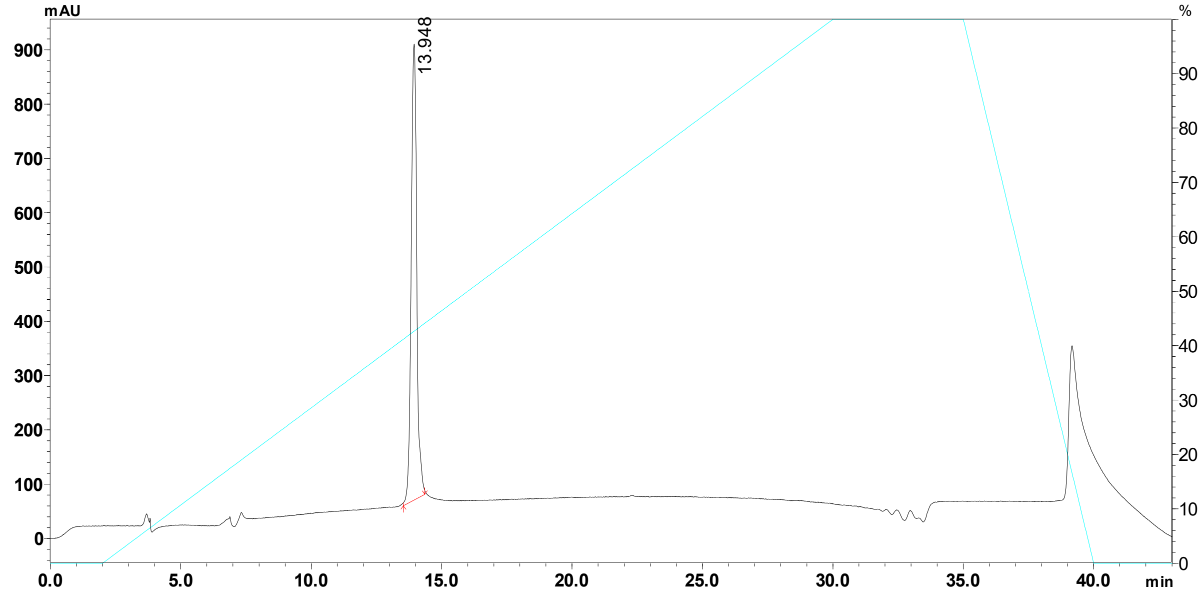

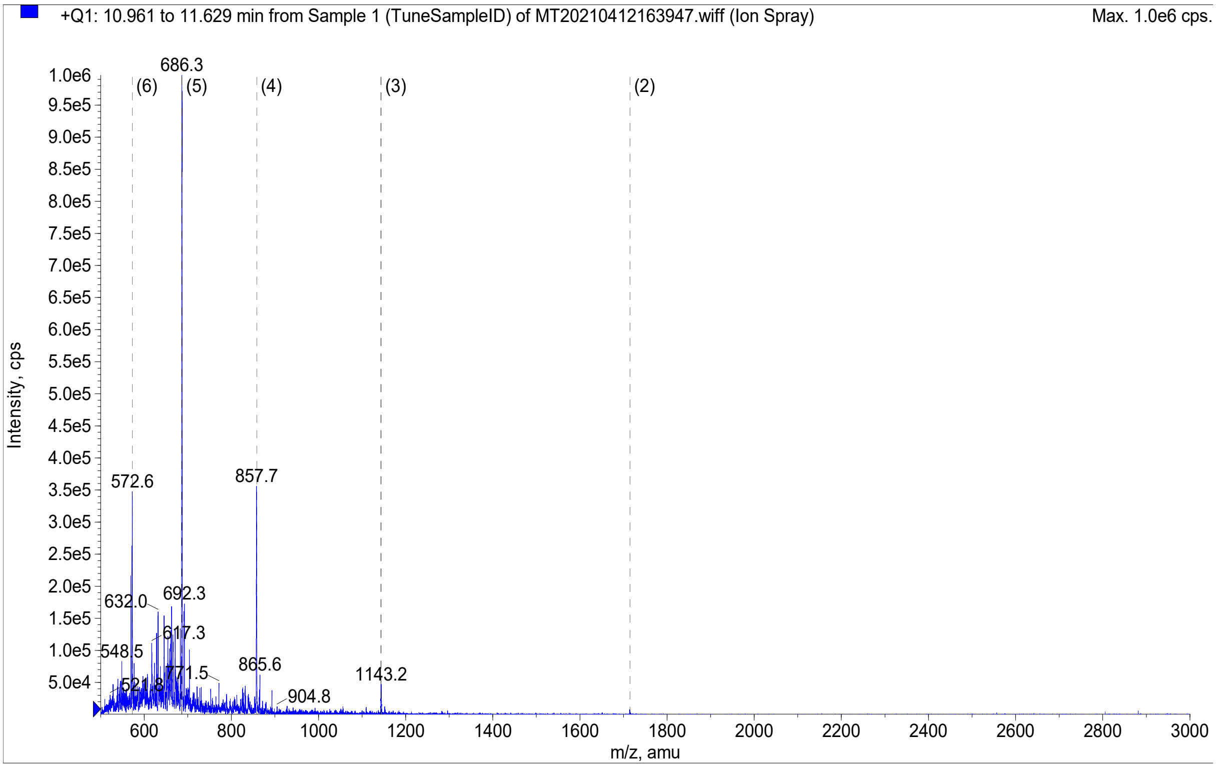

Figure S6. Analytical HPLC profile of J8-Cys (top); HPLC (C18): t*R* = 13.9 mins, purity ≥ 98%. Mol. Wt.= 3426.95g/mol. Mass spectrum of J8-Cys (bottom): $[M+3H]^{3+}$: 1143.2 (calculated 1143.3) , $[M+4H]^{4+}$: 857.7 (calculated 857.7), $[M+5H]^{5+}$: 686.3 (calculated 686.4), $[M+6H]^{6+}$: 572.6 (calculated 572.2); yield: 56%

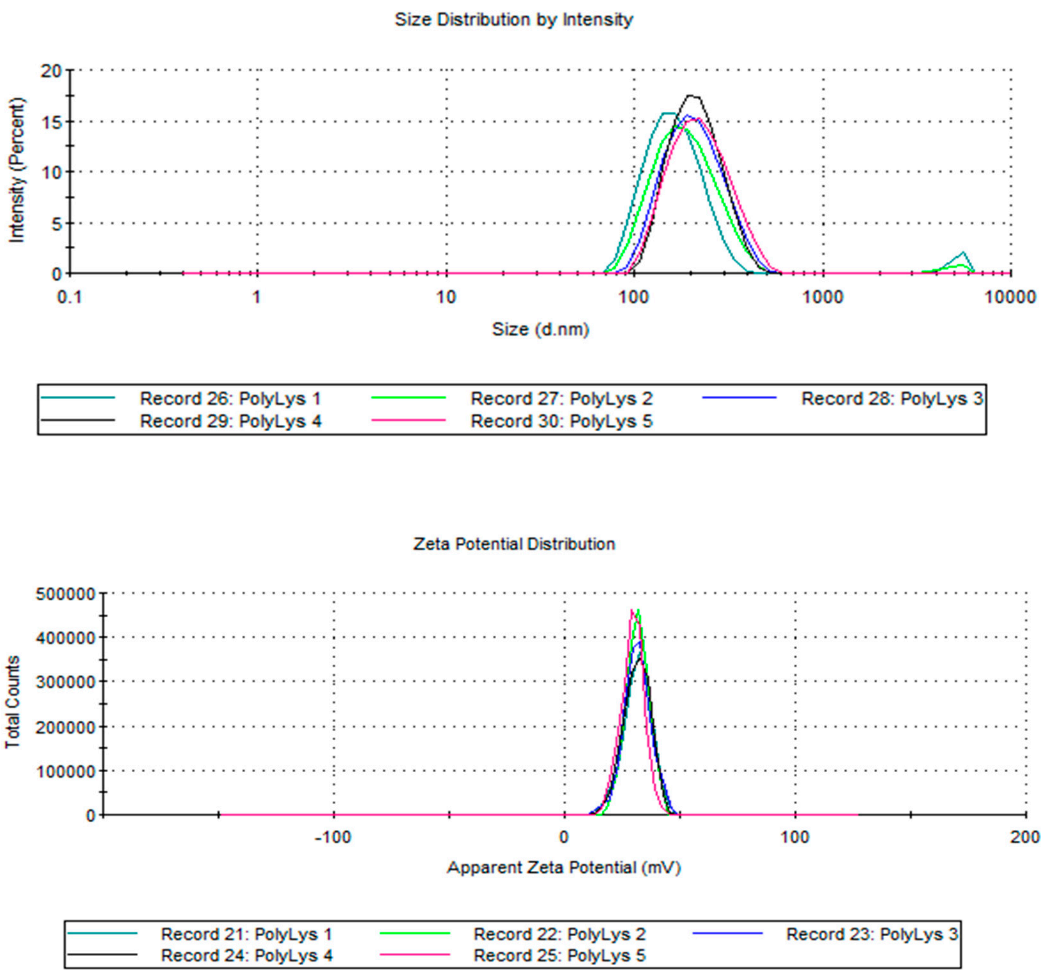

Figure S7. PEC-4: LCP-1/alginate/cross-linked poly-Lys nanocomplex; optimised concentration: 60µg. All measurements were performed using the Zetasizer software (Malvern Instrument, UK). Size distribution (top): 191±16, polydispersity index (PDI): 0.167 ± 0.05, zeta potential (bottom): 31.1±1.2

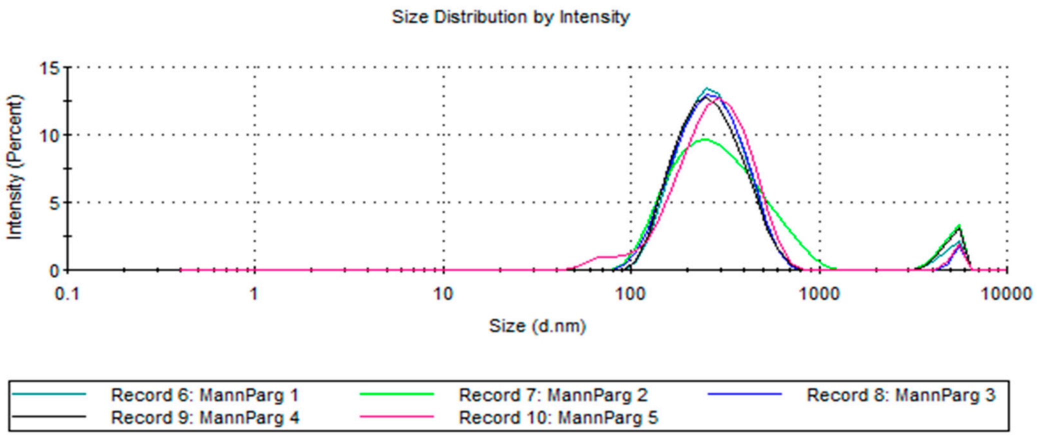

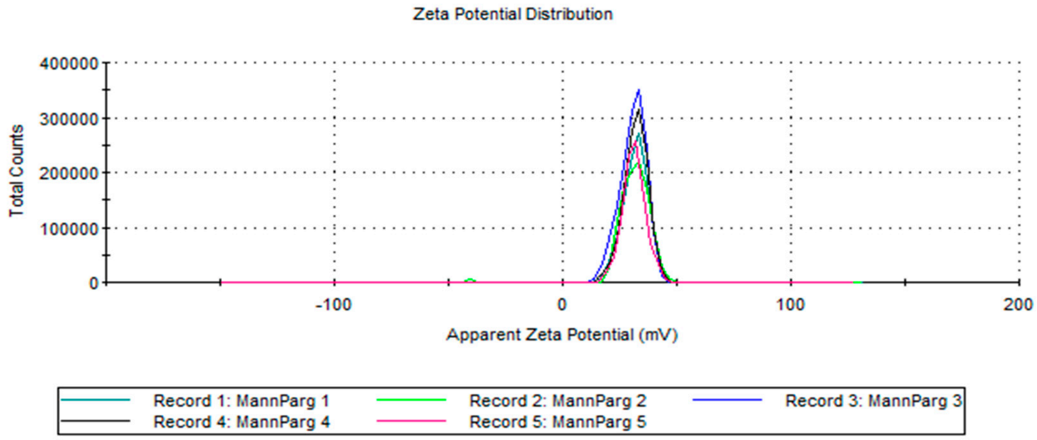

Figure S8. PEC-3: LCP-1/alginate/cross-linked mannosylated poly-Arg nanocomplex; optimised concentration: 90µg. All measurements were performed using the Zetasizer software (Malvern Instrument, UK). Size distribution (top): 286±10, polydispersity index (PDI): 0.335 ± 0.04, zeta potential (bottom): 31.5±0.5

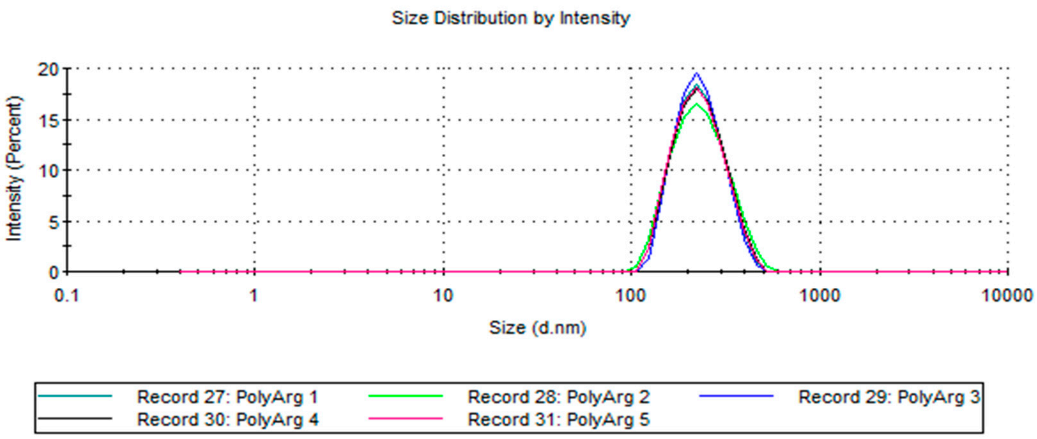

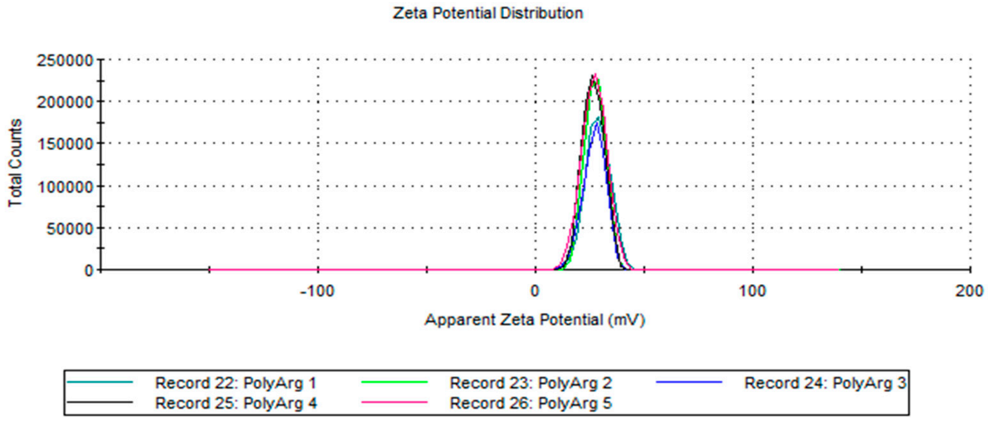

Figure S9. **PEC-2:** LCP-1/alginate/cross-linked poly-Arg; optimised concentration: 70µg. All measurements were performed using the Zetasizer software (Malvern Instrument, UK). Size distribution (top): 217±1, polydispersity index (PDI): 0.102 ± 0.01, zeta potential (bottom): 27.3± 0.8

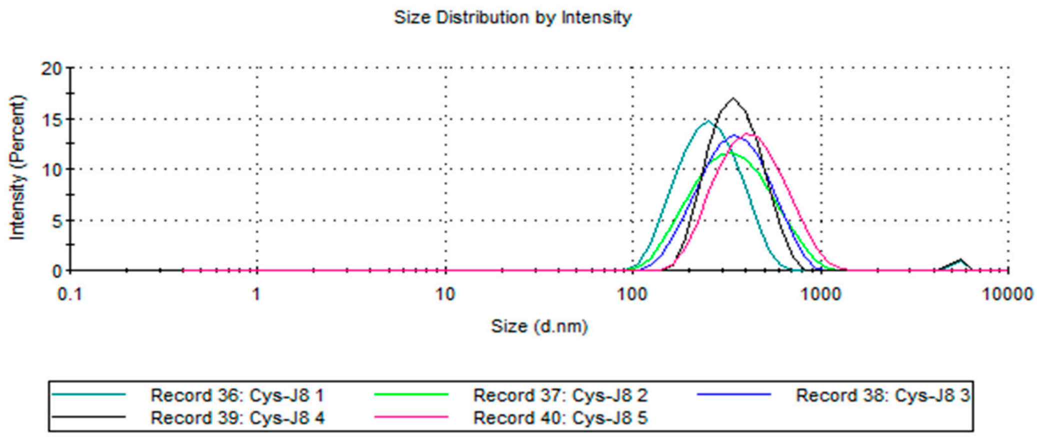

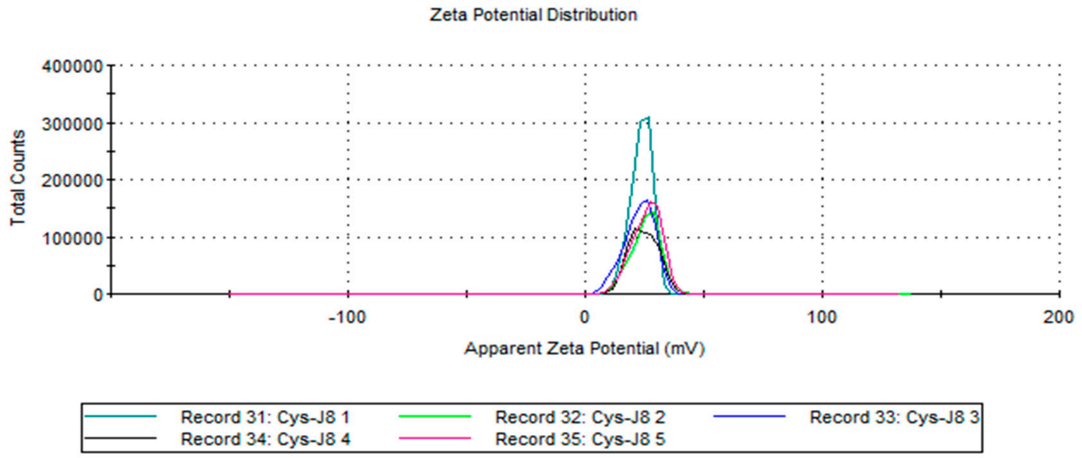

Figure S10. PEC-1: LCP-1/alginate/cross-linked J8-Cys-poly-Arg; optimised concentration: 110µg. All measurements were performed using the Zetasizer software (Malvern Instrument, UK). Size distribution (top): 330±56, polydispersity index (PDI): 0.194 ± 0.02, zeta potential (bottom): 24.4±1.2

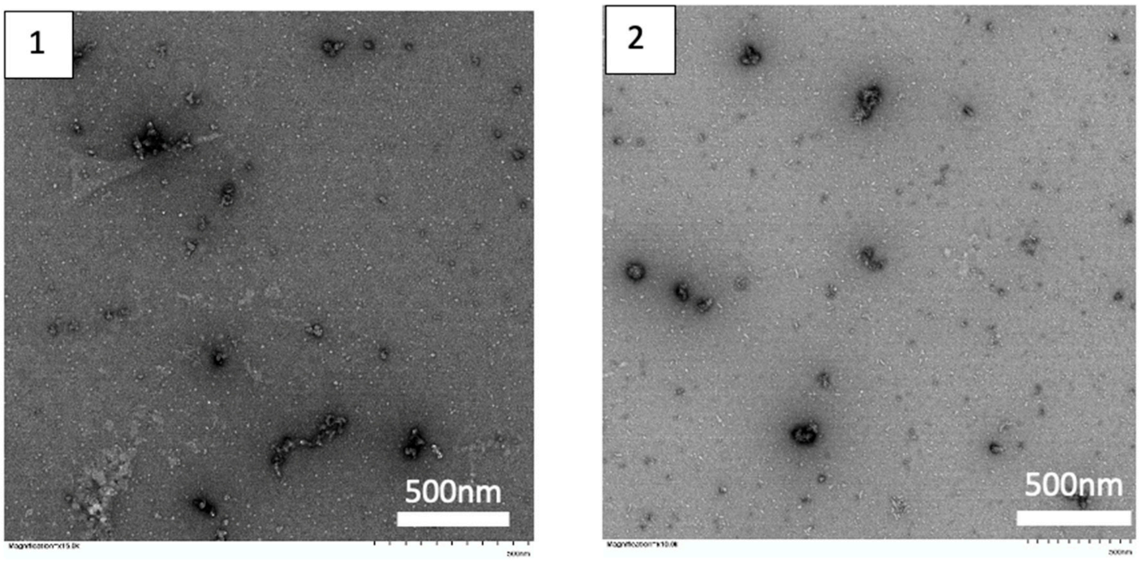

Figure S11. Transmission electron micrograph of (1) **PEC-2** and (2) **PEC-4** stained with 2% uranyl acetate (bar = 500 nm).

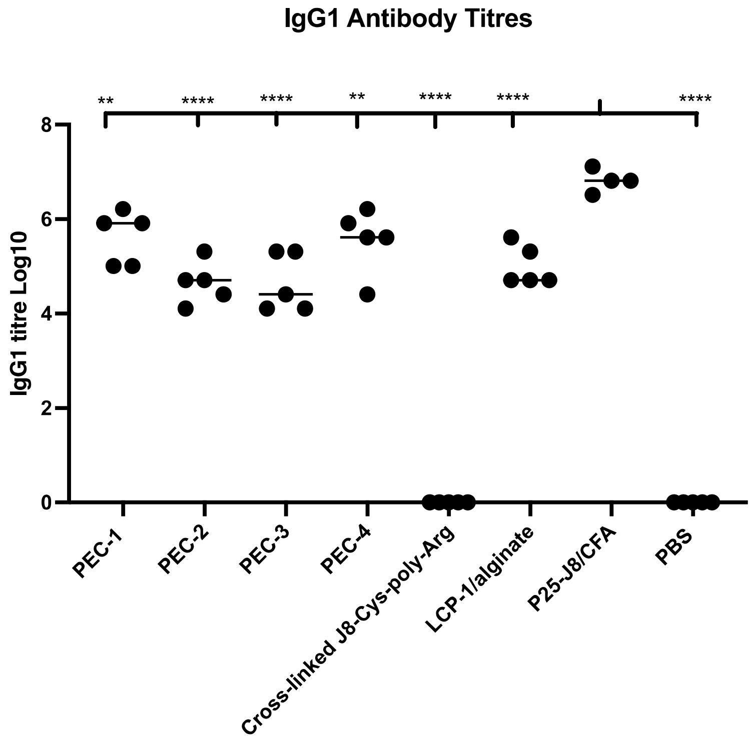

Figure S12. J8-specific IgG1 antibody titres following subcutaneous injections in BALB/c mice (n = 5), following immunization with vaccine candidates **PEC-0** to **PEC-4** and controls includes poly-Arg-J8, LCP-1/alginate and P25-CFA. Bars represents the average antigen-specific antibody titres. (ns: P > 0.05, *: P < 0.05, **: P < 0.01, ***: P < 0.001, ****: P < 0.0001)

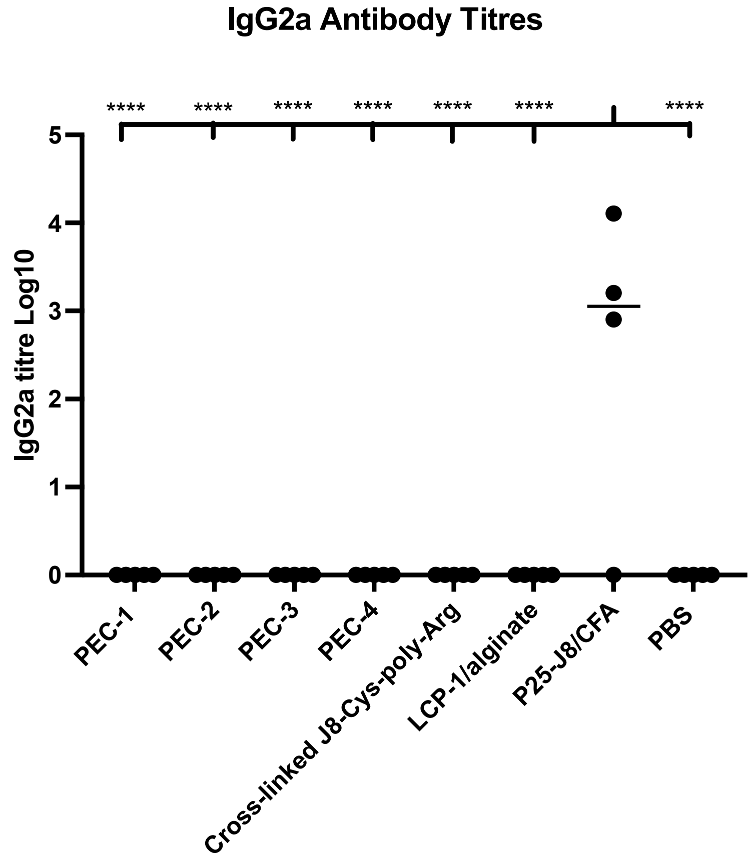

Figure S13. J8-specific IgG2a antibody titres following subcutaneous injections in BALB/c mice (n = 5), following immunization with vaccine candidates **PEC-0** to **PEC-4** and controls includes poly-Arg-J8, LCP-1/alginate and P25-CFA. Bars represents the average antigen-specific antibody titres. (ns: P > 0.05, *: P < 0.05, **: P < 0.01, ***: P < 0.001, ****: P < 0.0001)
